# Age demonstrates limited predictive utility for functional outcomes after thrombectomy in patients aged ≥70 years with acute ischemic stroke: a single-center cohort study

**DOI:** 10.3389/fneur.2026.1772084

**Published:** 2026-04-02

**Authors:** Pao-Sheng Yen, Victor C. Kok, Yu-Hui Lin, Li-Ying Ko

**Affiliations:** 1 Department of Neuroradiology, Kuang Tien General Hospital, Taichung, Taiwan; 2 Department of Nursing, Hungkuang University, Taichung, Taiwan; 3 Department of Internal Medicine, Kuang Tien General Hospital, Taichung, Taiwan; 4 Department of Neurology, Kuang Tien General Hospital, Taichung, Taiwan

**Keywords:** acute ischemic sroke, Bayesian logistic regression, endovascular thrombectomy (EVT), modified Rankin Scale (mRS), octo- and nonagenarians, older adults, post-thrombectomy outcomes

## Abstract

**Introduction:**

The clinical factor impact on outcomes after endovascular thrombectomy (EVT) in patients aged ≥70 years remains incompletely understood. We aimed to identify predictors of good outcomes after EVT in patients aged ≥70 years, defined as a modified Rankin Scale (mRS) score of 0–2.

**Methods:**

This retrospective, single-center cohort study included 94 patients aged ≥70 years with acute ischemic stroke who underwent EVT. Participants were stratified into septuagenarians (*n* = 44) and octo/nonagenarians (*n* = 50). We evaluated post-EVT modified thrombolysis in cerebral infarction reperfusion grade, symptomatic intracerebral hemorrhage, and mRS score at 3 months follow-up as outcomes. Both multivariable (LR) and Bayesian logistic regression (BLR) and sensitivity analyses were conducted to derive adjusted odds ratio (aOR) and assess the probabilistic associations between clinical variables and outcomes.

**Results:**

At presentation, the median ischemic core was higher in octo/nonagenarians compared to septuagenarians (20 mL vs. 4 mL, *p* = 0.0464); median Alberta Stroke Program Early CT Score was lower (7 vs. 8, *p* = 0.0112). Higher Fazekas grades of leukoaraiosis were more frequent in octo/nonagenarians (*p* = 0.0297) than in septuagenarians. Good mRS outcomes were achieved by 27.3% of septuagenarians vs. 8.0% of octo/nonagenarians (*p* = 0.0274). In the multivariable LR, age was not an independent predictor of poor outcomes (aOR 2.40; 95% CI, 0.65–10.08; *p* = 0.1991). BLR identified higher National Institutes of Health Stroke Scale scores [odds ratio (OR) 0.90; 95% credible interval, 0.81–0.98] associated with poorer outcomes, whereas prior intravenous thrombolysis (OR 6.59; 1.16–23.09) predicted better outcomes. BLR did not show probabilistic certainty of age as a predictor of functional outcomes. Including infarct core in the model did not impact sensitivity analysis results.

**Conclusion:**

Age was not independently associated with functional outcomes. Age-related differences in outcomes may be mediated by initial stroke characteristics rather than age.

**Clinical trial registration:**

ClinicalTrials.gov, identifier NCT06953427.

## Graphical abstract

**Figure:**
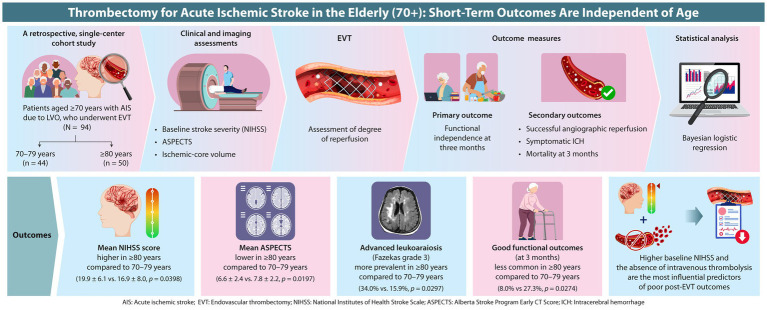

## Introduction

Endovascular intervention for patients with acute ischemic stroke (AIS) includes either mechanical thrombectomy or intra-arterial thrombolysis combined with routine medical treatment. A recent Cochrane systematic review of 18 randomized controlled trials (RCTs) showed with high-certainty evidence that endovascular intervention increases the likelihood of achieving a favorable functional outcome [modified Rankin Scale (mRS) score of 0–2] by 50% in patients with AIS (1). Furthermore, a cross-Atlantic RCT was conducted to determine whether endovascular therapy (EVT) plus routine medical care is superior to medical care alone in patients with acute proximal cerebral vessel occlusion in anterior circulation and large infarcts, regardless of infarct size. The study confirmed a 63% increase in odds of a favorable outcome with EVT plus medical care (2). Notably, accumulating data indicate that pediatric patients may also benefit from EVT, demonstrating high recanalization rates and favorable neurological outcomes (3).

In real-world registries of EVT for AIS due to large-vessel occlusion (LVO) in anterior circulation, approximately 50% of patients are aged ≥70 years (4), 13–39% are aged ≥80 years (5–8), and 6–8% are aged ≥90 years (Supplementary Table S1) (9, 10). In the Canadian Quality Improvement and Clinical Research registry, 50% of patients undergoing EVT were aged ≥70 years, with 25% aged ≥80 years (4). Similarly, the Netherlands MR CLEAN registry reported that 25% of EVT-treated patients were ≥80 years old (7). An updated report of the Netherlands study revealed that the median age of patients with anterior circulation AIS enrolled in the study increased from 66 years in 2014 to 74 years in 2018 (11). A seminal clinical trial (DAWN) demonstrated that among patients with ischemic stroke last known to be well 6 to 24 h earlier, endovascular thrombectomy (EVT) was associated with significantly better functional outcomes than standard medical care alone—even in older adults. Notably, 26.2% of the cohort were aged ≥80 years (*n* = 54), and the benefit of EVT extended to this subgroup (12). However, subsequent real-world studies in octogenarian populations have yielded mixed results. A recent UK single-center study evaluating EVT techniques in anterior circulation ischemic stroke (ACIS) reported that 21.1% of patients (*n* = 105) were aged ≥80 years. In this cohort, thromboaspiration (TAS) alone—used in 54.3% of cases—was identified as the strongest predictor of favorable outcomes in the elderly, compared to stent retriever (SR) or combined TAS + SR techniques, which were employed in only 27.6% of cases. Nonetheless, functional outcomes in this study remained significantly worse in patients aged ≥80, with 81% having a 90-day modified Rankin Scale (mRS) > 2, compared to 54.8% in younger patients (13). Similarly, a U.S. nationwide claims-based study, in which 25% of participants were aged ≥80 years, found that only 9% of these older patients achieved a good outcome, defined by discharge to home or facilities requiring minimal assistance (14). Another large multicenter real-world study also reported substantially lower rates of functional independence in octogenarians undergoing EVT, compared to their younger counterparts (15). These findings underscore the need for further investigation into EVT outcomes in this vulnerable elderly population.

Clinicians are now more willing to undertake the challenges of performing EVT in much older patients and are approaching these cases with greater assertiveness. In patients aged ≥70 years who are EVT-eligible, post-procedure mortality increases progressively with increasing age. A prospective European study found that each additional year of age was associated with an 8% decline in the likelihood of achieving a favorable functional outcome (6). In older patients, increasing age is more than just a number—it reflects a higher likelihood of significant medical comorbidities, polypharmacy, declining functional status, compromised nutritional status, and weakened immune function.

Studies regarding age as a predictor of EVT outcomes often compare older patients to much younger counterparts. However, contrasting post-EVT outcomes in older adults aged ≥80 years with those of adults aged <70 years is neither realistic nor appropriate due to inherent differences in baseline health, comorbidities, and physiological resilience. We hypothesized that advanced age (≥80 years), independent of other clinical factors, is associated with poorer short-term functional outcomes following EVT. To test this hypothesis, we conducted both multivariable logistic regression and Bayesian logistic regression (BLR) analyses of older patients aged ≥70 years from our prospectively maintained intervention neuroradiology stroke registry and compared their post-EVT functional outcomes to those of septuagenarians (aged 70–79 years), selected as a more clinically comparable control group.

## Materials and methods

### Study design and population

This was a retrospective, single-center cohort study evaluating patients aged ≥70 years with AIS due to LVO, who underwent EVT between 2021 and 2023 at Kuang Tien General Hospital. This stroke center in central Taiwan’s west-coast region is nationally accredited for its capability to evaluate and perform EVT for AIS emergencies (16, 17). The study was approved by the Kuang Tien General Hospital Institutional Review Board (approval number: KTGH-11415), and the requirement for informed consent was waived due to the retrospective nature of the study. This study was also registered on April 16, 2025 at ClinicalTrials.gov (Identifier: NCT06953427).

### Inclusion and exclusion criteria

**Figure 1:**
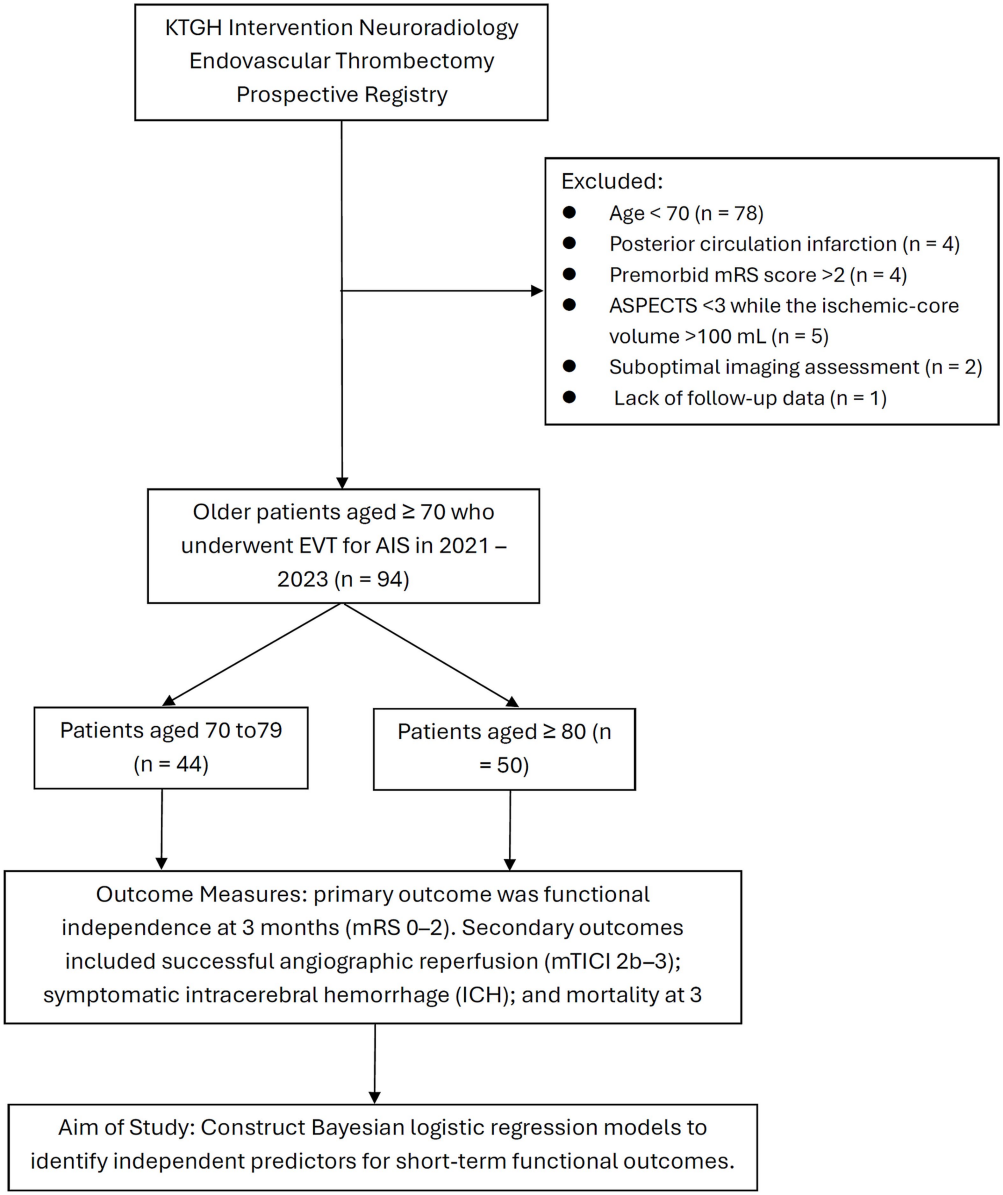
Study flow diagram of the retrospective analysis based on a prospectively maintained registry.

We included patients as follows: age ≥70 years; AIS due to anterior circulation proximal cerebral artery (intracranial ICA, MCA M1, and ACA A1 segments) LVO confirmed by computed tomography (CT) angiography, consistent with the patient’s stroke symptoms; severity of stroke documented with the National Institutes of Health Stroke Scale (NIHSS) score between 6 and 30; premorbid mRS score ≤2; CT perfusion (CTP) imaging indicating a mismatch ratio >1.8, alongside an ischemic-core volume <100 mL; CT scan confirming the absence of intracranial hemorrhage; EVT procedure performed within 24 h from the time last seen well; and available mRS score at 3-month follow-up. We excluded patients with posterior circulation infarction; premorbid mRS score ≥3; Alberta Stroke Program Early CT Score (ASPECTS) < 3 and ischemic-core volume >100 mL; and poor imaging quality precluding assessment or lack of follow-up data. Patients were stratified into two groups: septuagenarians, 70–79 years; and octogenarians and nonagenarians, ≥80 years (Figure 1).

### Assessment and endovascular procedures

Baseline stroke severity was assessed using the NIHSS (18). Patients presenting within 3 h of symptom onset were treated with intravenous recombinant tissue plasminogen activator (IV thrombolysis), if eligible, in accordance with our institutional AIS protocol (19). Non-contrast CT and CTP were used to evaluate the ASPECTS and the ischemic-core volume (20). Following standard angiographic techniques, EVT was performed using stent retrievers and/or aspiration catheters. Most patients underwent thrombectomy using a combined TAS + SR approach. The degree of reperfusion was graded using the modified Thrombolysis in Cerebral Infarction (mTICI) scale (21), with mTICI 2b–3 defined as successful reperfusion. All patients underwent MRI studies 1 day after the procedure. The MRI analyses were conducted independently by an experienced neurologist and a neuroradiologist (YHL, PSY), blinded to clinical information, using the Fazekas scale to assess the extent of leukoaraiosis in the contralateral asymptomatic hemisphere only, thereby avoiding the confounding effects of acute ischemic lesions. Any disagreements were resolved through consensus discussion between the two raters (22).

### Outcome measures

The primary outcome was functional independence at 3 months (mRS 0–2) (23). Secondary outcomes included successful angiographic reperfusion (mTICI 2b–3); symptomatic intracerebral hemorrhage (ICH); and mortality at 3 months (mRS score 6).

### Statistical analysis

Baseline characteristics and outcomes were compared between age groups. Continuous variables were analyzed using the t-test or Mann–Whitney U test, and categorical variables using the *χ*^2^ or Fisher’s exact test. We constructed univariate logistic regression (LR) models to evaluate the association between a good short-term outcome—defined as a modified Rankin Scale (mRS) score of 0 to 2—and a set of explanatory variables (X₁, X₂, X₃, …, X_n_). Initially, univariate logistic regression analyses were performed for each explanatory variable. Variables with a *p*-value < 0.05 in the univariate analyses were considered for inclusion in the multivariable LR and the BLR model. Given the limited sample size and relatively low event rate in this rare, older stroke population, BLR was employed to enhance the stability of effect estimates. This approach allowed the incorporation of prior clinical evidence—such as the known prognostic value of NIHSS, ASPECTS, and intravenous thrombolysis—to inform the model through weakly informative priors. Bayesian inference further enabled probabilistic interpretation of model parameters, supporting more nuanced clinical decision-making in this high-risk subgroup. BLR was conducted using StataNow 19.5. Default noninformative priors were applied, specifying independent normal distributions for all regression coefficients: Normal (0, 10^6^), as implemented in Stata’s bayes: logit procedure. Markov Chain Monte Carlo sampling was performed using the random-walk Metropolis–Hastings algorithm with 12,500 iterations, including a burn-in of 2,500. A total of 10,000 posterior samples were retained for inference.

To address potential multicollinearity among predictors, we assessed the variance inflation factor (VIF) after including multiple candidate variables in the model, prior to conducting the final BLR analysis. VIF values for all selected predictors in the main BLR were below 1.25, well below the stringent threshold of 2, indicating acceptable levels of collinearity among predictors. Finally, as a sensitivity analysis, we also constructed an alternative BLR model that included variables with a *p*-value < 0.10 in the univariate analysis, to assess the robustness of the findings and account for potential confounders that may have been excluded under the stricter threshold. All statistical analyses were performed on either R version 4.2.1. (R Foundation for Statistical Computing, Vienna, Austria) or StataNow 19.5 Basic Edition (StataCorp, College Station, TX). StataNow was used for BLR. A *p*-value < 0.05 was considered statistically significant in non-Bayesian statistics.

## Results

### Patient characteristics

A total of 94 patients aged ≥70 years who underwent EVT for AIS were included, with 44 (46.8%) in the septuagenarian group (70–79 year-old) and 50 (53.2%) in the octo/nonagenarian group (≥80 to 99 year-old). The octogenarian/nonagenarian group demonstrated a trend toward a higher baseline NIHSS score, with a median (IQR) of 21 (17–24), compared to the septuagenarian group, whose median score was 19 (9.75–22) (*p* = 0.0619). Additionally, the older cohort had a significantly lower median ASPECTS score of 7 (5–8.75) versus 8 (7–9) in the septuagenarians (*p* = 0.0112) (Table 1). The older cohort also had a larger infarct core volume on CT perfusion, with a median of 20 mL (IQR, 1–53.25 mL), compared to 4 mL (IQR, 0–26 mL) in the septuagenarian group (*p* = 0.0464). Advanced leukoaraiosis (Fazekas grade 3) was more prevalent among octo/nonagenarians (34.0% vs. 15.9%, *p* = 0.0297) (Table 1). There were no statistically significant differences among the three age groups (by decade) regarding sex distribution, rates of hypertension, diabetes, atrial fibrillation, or baseline ischemic-core volume on CT perfusion (Supplementary Table S2).

**Table 1:** Demographic and clinical characteristics stratified by age group in patients aged ≥70 years.

Variable/characteristic	Age 70–79 years subgroup (*N* = 44)		Age ≥ 80 years subgroup (*N* = 50)		*p*-value
Sex					0.2457
Male (%)	22 (50%)		18 (36%)		
Female (%)	22 (50%)		32 (64%)		
NIHSS, initial					
Very severe (score >25)	6	13.6%	10	20%	0.5863
Severe stroke (score >14)	32	72.7%	44	88.0%	0.1063
Median (IQR)	19	(9.75–22)	21	(17–24)	0.0619
DTP time, (min)					
Median (IQR)	128	(57–165.25)	127.5	(93.75–162.75)	0.9125
PTR time, (min)					
Median (IQR)	30.5	(25–39)	33	(25.25–48.75)	0.2851
ASPECTS					
Median (IQR)	8	(7–9)	7	(5–8.75)	**0.0112**
CTP infarct core (mL)					
Large infarct core (≥70 mL)	4	9.1%	8	16%	0.5608
Median (IQR)	4	(0–26)	20	(1–53.25)	**0.0464**
Not obtainable	6	13.6%	4	8.0%	
Occlusion site					0.4312
A2	1	2.3%	0	0%	
ACA	1	2.3%	1	2.0%	
ICA	10	22.7%	17	34.0%	
M1	18	40.9%	17	34.0%	
M2	11	25.0%	9	18.0%	
Tandem lesions	3	6.8%	6	12.0%	
Hypertension					0.8700
Yes	35	79.5%	38	76.0%	
No	9	20.5%	12	24.0%	
Type 2 diabetes					1.0000
Yes	24	54.5%	27	54.0%	
No	20	45.5%	23	46.0%	
Dyslipidemia					0.1403
Yes	17	38.6%	28	56.0%	
No	27	61.4%	22	44.0%	
Atrial fibrillation					0.3888
Yes	28	63.6%	37	74.0%	
No	16	36.4%	13	26.0%	
Coronary artery disease					0.2619
Yes	16	36.4%	25	50.0%	
No	28	63.6%	25	50.0%	
Peripheral arterial obstructive disease					0.7261
Yes	5	11.4%	8	16.0%	
No	39	88.6%	42	84.0%	
Smoking					0.1482
Yes	9	20.5%	4	8.0%	
No	35	79.5%	46	92.0%	
Intravenous thrombolysis					0.2823
Yes	13	29.5%	9	18.0%	
No	31	70.5%	41	82.0%	
Family support					0.1306
Yes	36	81.8%	47	94.0%	
No	8	18.2%	3	6.0%	
Fazekas grading of leukoaraiosis					**0.0297**
0	5	11.4%	0	0%	
1	16	36.4%	18	36.0%	
2	16	36.4%	15	30.0%	
3	7	15.9%	17	34.0%	

Boldface *p*-values indicate statistical significance. ACA, anterior cerebral artery; ASPECTS, Alberta Stroke Program Early CT Score; CTP, computed tomography perfusion; DTP, door-to-puncture time; NIHSS, National Institutes of Health Stroke Scale. ICA, internal carotid artery; PTR, puncture-to-reperfusion time; SD, standard deviation.

### Procedural outcomes

Successful reperfusion (mTICI 2b–3) was achieved in 93.2% of patients, with no significant difference between septuagenarians (93.2%) and octo/nonagenarians (96.0%) (*p* = 0.4105) (Table 2). Median door-to-puncture and puncture-to-reperfusion times were comparable across age groups, suggesting no major procedural delays related to age (Supplementary Table S2). Rates of post-procedural symptomatic ICH were similar (22.7% vs. 20.0%, *p* = 0.9443) (Table 2).

**Table 2:** Outcomes post-endovascular thrombectomy (EVT) compared by age group.

Outcome	Age 70–79 years (*N* = 44)		Age ≥ 80 years (*N* = 50)		*p*-value
Post-EVT TICI					0.4105
0	1	2.3%	0	0%	
1	1	2.3%	2	4.0%	
2a	1	2.3%	0	0%	
2b	15	34.1%	20	40.0%	
2c	8	18.2%	3	6.0%	
3	18	40.9%	25	50.0%	
Symptomatic ICH					0.9443
Yes	10	22.7%	10	20.0%	
No	34	77.3%	40	80.0%	
mRS at 3 months follow-up					**0.0347**
0	1	2.3%	2	4.0%	
1	4	9.1%	2	4.0%	
2	7	15.9%	0	0%	
3	7	15.9%	9	18.0%	
4	11	25.0%	12	24.0%	
5	7	15.9%	6	12.0%	
6	7	15.9%	19	38.0%	
mRS grouped by outcome					**0.0274**
0–2 (Good)	12	27.3%	4	8.0%	
3–6 (Intermediate to Poor)	32	72.7%	46	92.0%	

Boldface *p*-values indicate statistical significance.

ICH, intracerebral hemorrhage; mRS, modified Rankin Scale; TICI, Thrombolysis in Cerebral Infarction grading system.

### Functional and mortality outcomes

At 3 months, good functional outcomes (mRS 0–2) were significantly more common in septuagenarians compared to octo/nonagenarians (27.3% vs. 8.0%, *p* = 0.0274) (Table 2). The mortality rate (mRS 6) was higher in the older group (38.0% vs. 15.9%, *p* = 0.0347) (Table 2).

In univariate logistic regression analysis, older age (≥80 years) was associated with significantly higher odds of poor functional outcome [odds ratio (OR) 4.31; 95% confidence interval (CI), 1.37–16.54; *p* = 0.0187] (Table 3). However, in the multivariable LR, age was not an independent predictor of poor outcomes [adjusted OR (aOR) 2.40; 95% CI, 0.65–10.08; *p* = 0.1991] (Table 4). Instead, higher NIHSS at presentation (aOR 1.10; 95% CI, 1.01–1.21; *p* = 0.0343) and lack of intravenous thrombolysis (aOR 0.24; 95% CI, 0.06–0.92; *p* = 0.0395) were significant predictors of poor functional outcomes (Table 4).

**Table 3:** Comparison of clinical and imaging characteristics between patients with/without good outcomes in terms of mRS at 3 months after endovascular thrombectomy.

Characteristic	Good outcome (mRS 0–2) (*N* = 16)		Poor outcome (mRS 3–6) (*N* = 78)		Univariate analysis		
	*N*	%/IQR	*N*	%/IQR	OR	95% CI	*p*-value
Age (years)							
70–79	12	75.0%	32	41.0%	–	–	–
80+	4	25.0%	46	59.0%	4.31	(1.37–16.54)	**0.0187**
Sex							
Female	4	25.0%	36	46.2%	–	–	–
Male	12	75.0%	42	53.8%	2.57	(0.81–9.83)	0.1279
NIHSS							
Median (IQR)	15.5	(9.75–19)	20.5	(17–24)	1.10	(1.02–1.18)	**0.0144**
Door-to-Puncture Time (min)							
Median (IQR)	16	139.5 (121.5–165.5)	78	125 (54.75–163.75)	1.00	(1.00–NA)	0.6170
Puncture-to-Reperfusion Time (min)							
Median (IQR)	16	32 (26.5–41.25)	78	32 (25–48)	1.00	(0.98–1.02)	0.9945
ASPECTS							
Median (IQR)	8.5	(7.75–10)	7	(5–9)	0.67	(0.46–0.90)	**0.0189**
CTP infarct core (mL)							
Median (IQR)	4	(0–12)	17	(0–50)	1.03	(1.00–1.06)	0.0777
Occlusion site							
A2	0	0.0%	1	1.3%	–	–	–
ACA	0	0.0%	2	2.6%	1.00	(0.00–Inf)	1.0000
ICA	3	18.8%	24	30.8%	0.00	(NA–Inf)	0.9980
M1	8	50.0%	27	34.6%	0.00	(NA–Inf)	0.9980
M2	5	31.3%	15	19.2%	0.00	(NA–Inf)	0.9980
Tandem	0	0.0%	9	11.5%	1.00	(0.00–Inf)	1.0000
Post-EVT TICI							
0	0	0.0%	1	1.3%	–	–	–
1	0	0.0%	3	3.8%	1.00	(0.00–Inf)	1.0000
2a	0	0.0%	1	1.3%	1.00	(0.00–Inf)	1.0000
2b	2	12.5%	33	42.3%	0.00	(NA–Inf)	0.9970
2c	1	6.3%	10	12.8%	0.00	(NA–Inf)	0.9970
3	13	81.3%	30	38.5%	0.00	(NA–Inf)	0.9970
Hypertension							
Yes	11	68.8%	62	79.5%	1.76	(0.50–5.63)	0.3517
No	5	31.3%	16	20.5%	–	–	–
Type 2 diabetes							
Yes	10	62.5%	41	52.6%	0.66	(0.21–1.97)	0.4690
No	6	37.5%	37	47.4%	–	–	–
Dyslipidemia							
Yes	6	37.5%	39	50.0%	1.67	(0.56–5.32)	0.3650
No	10	62.5%	39	50.0%	–	–	–
Atrial fibrillation							
Yes	11	68.8%	54	69.2%	1.02	(0.30–3.15)	0.9697
No	5	31.3%	24	30.8%	–	–	–
Coronary artery disease							
Yes	5	31.3%	36	46.2%	1.89	(0.62–6.45)	0.2780
No	11	68.8%	42	53.8%	–	–	–
Peripheral arterial obstructive disease							
Yes	2	12.5%	11	14.1%	1.15	(0.27–7.95)	0.8660
No	14	87.5%	67	85.9%	–	–	–
Cigarette smoking							
Yes	3	18.8%	10	12.8%	0.64	(0.17–3.12)	0.5340
No	13	81.3%	68	87.2%	–	–	–
Symptomatic ICH							
Yes	1	6.3%	19	24.4%	4.83	(0.89–90.19)	0.1400
No	15	93.8%	59	75.6%	–	–	–
Intravenous thrombolytic therapy							
Yes	7	43.8%	15	19.2%	0.31	(0.10–0.98)	**0.0413**
No	9	56.3%	63	80.8%	–	–	–
Family support							
Yes	14	87.5%	69	88.5%	1.10	(0.16–4.85)	0.9132
No	2	12.5%	9	11.5%	–	–	–
Fazekas grading of leukoaraiosis							
0	1	6.3%	4	5.1%	–	–	–
1	6	37.5%	28	35.9%	1.17	(0.05–9.87)	0.8980
2	5	31.3%	26	33.3%	1.30	(0.06–11.48)	0.8300
3	4	25.0%	20	25.6%	1.25	(0.06–11.86)	0.8580

Boldface *p*-values indicate statistical significance. ACA, anterior cerebral artery; ASPECTS, Alberta Stroke Program Early Computed Tomography Score; CTP, computed tomography perfusion; NIHSS, National Institutes of Health Stroke Scale. ICA, internal carotid artery.

**Table 4:** Multivariable logistic regression model derived odds ratios of predictive factors for poor functional outcome in terms of mRS at 3 months after EVT (poor outcome = 1; good outcome = 0).

Characteristics	Crude OR			Adjusted OR		
	OR	95% CI	*p*-value	aOR	95% CI	*p*-value
Age						
70–79	Reference			Reference		
80+	4.31	(1.37–16.54)	**0.0187**	2.40	(0.65–10.08)	0.1991
NIHSS						
	1.10	(1.02–1.18)	**0.0144**	1.10	(1.01–1.21)	**0.0343**
ASPECTS						
	0.67	(0.46–0.90)	**0.0189**	0.76	(0.50–1.09)	0.1738
Intravenous thrombolytic therapy						
Yes	0.31	(0.10–0.98)	**0.0413**	0.24	(0.06–0.92)	**0.0395**
No	Reference			Reference		

Bold typeface denotes statistical significance.

aOR, adjusted odds ratio; ASPECTS, Alberta Stroke Program Early CT Score; CI, confidence interval; mRS, modified Rankin scale; NIHSS, National Institutes of Health Stroke Scale; OR, odds ratio; SD, standard deviation.

Furthermore, in BLR, age was not a principal predictor of good functional outcomes [posterior mean OR 0.95; 95% Credible Interval (CrI), 0.86–1.05] (Table 5). BLR identified baseline NIHSS score as negatively associated with good functional outcome (posterior mean OR, 0.90; 95% CrI, 0.81–0.98), indicating a high posterior probability that greater stroke severity reduces the likelihood of favorable recovery. In contrast, prior intravenous thrombolysis was positively associated with good outcome (posterior mean OR, 6.59; 95% CrI, 1.16–23.09), suggesting a strong probabilistic benefit (Table 5; Supplementary Table S4). A sensitivity analysis was conducted using BLR to assess good functional outcomes, defined as a mRS score at 3 months (good outcome = 1; poor outcome = 0), with adjustment for ischemic-core volume as a potential confounder (Supplementary Table S3). The inclusion of infarct core volume in sensitivity analyses did not substantially alter these associations.

**Table 5:** Bayesian logistic regression model in predicting good functional outcome in terms of mRS (0–2) at 3 months after endovascular thrombectomy.

Predictor	Odds ratio	Std. dev.	95% CrI	Median	MCSE
Age	0.95*	0.05	0.86–1.05	0.95	0.002
Intravenous thrombolysis	6.59	5.62	1.16–23.09	4.72	0.43
NIHSS	0.90	0.04	0.81–0.98	0.90	0.00
ASPECTS	1.44*	0.30	0.97–2.08	1.40	0.02

*Because the 95% credible interval (CrI) includes 1, the posterior distribution indicates non-negligible uncertainty regarding the effect.

ASPECTS, Alberta Stroke Program Early CT Score; CrI, credible interval; MCSE, Monte Carlo Standard Error; mRS, modified Rankin Scale; NIHSS, National Institutes of Health Stroke Scale; OR, odds ratio; Std. dev., posterior standard deviation of the estimated parameter.

## Discussion

EVT has been firmly established as the standard of care for AIS due to LVO, even among older adult patients. However, the extent to which age independently influences post-EVT outcomes remains an area of debate. In this single-center analysis, although octo- and nonagenarians demonstrated worse unadjusted functional and mortality outcomes compared to septuagenarians, age was not an independent predictor in the multivariable logistic regression analysis, nor did it show probabilistic certainty as a predictor of functional outcomes in the Bayesian logistic regression model. Instead, higher stroke severity at presentation (NIHSS) and the absence of intravenous thrombolysis were the strongest determinants of poor prognosis. These findings contribute to the growing body of evidence suggesting that chronological age alone should not preclude EVT consideration in appropriately selected older adult patients. A recent meta-analysis comparing direct endovascular thrombectomy (DEVT) with bridging therapy demonstrated that the combined use of intravenous thrombolysis followed by EVT is associated with improved functional outcomes without a significant increase in symptomatic intracranial hemorrhage compared with direct EVT alone (24).

Although age was not found to be a principal predictor of short-term functional outcomes after EVT, older patients are more likely to present with higher NIHSS scores and lower ASPECTS. As these unfavorable factors tend to cluster with advancing age, clinicians may still observe poorer outcomes among much older adults, reinforcing the need for careful patient selection rather than age-based exclusion. The lack of predictive power for age in the classical LR and BLR models likely reflects the fact that worse baseline characteristics—such as higher NIHSS, lower ASPECTS, greater white matter disease burden (Fazekas), and larger CTP core volumes—mediate the apparent effect of age. In essence, patients aged 80 and above tend to present in a sicker state, and these factors are the true drivers of outcome. The paradox of age and EVT outcomes remains interesting. Despite the well-documented increase in mortality and disability with advancing age, our study reinforces the notion that age itself may not be the primary determinant of EVT success. This aligns with prior studies, including the MR CLEAN Registry and the HERMES meta-analysis, both of which demonstrated that EVT remains beneficial across all age groups, albeit with declining rates of functional independence in older patients (7, 25). Similarly, a large meta-analysis confirmed that although EVT outcomes were less favorable in older patients, the benefit of EVT persisted in those aged ≥80 years compared to standard medical therapy alone (26). One explanation for this paradox lies in the concept of selective survivorship and pre-stroke functional status. Many older patients undergoing EVT are functionally independent at baseline and have already survived other cerebrovascular risk factors, suggesting a degree of physiological resilience. Conversely, frail older adults with substantial pre-stroke disability may not have been offered EVT or were excluded from analysis, leading to a selection bias in favor of more robust older patients. Ippen et al. (10) demonstrated that premorbid functional status, as assessed by the mRS, had a stronger impact on EVT outcomes than age alone, reinforcing the importance of pre-stroke independence in clinical decision-making (10, 27). Importantly, emerging neuroimaging evidence suggests that biological brain age—quantified as the brain age gap—may more accurately capture underlying cerebral vulnerability and resilience than chronological age alone, as longitudinal data demonstrate that an older-appearing brain predicts subsequent cognitive decline after stroke whereas chronological age does not and that a higher baseline brain age gap is associated with increased risk of post-stroke neurocognitive disorder over time, collectively supporting biological brain age as a more explanatory determinant of functional neurological outcomes following EVT (28, 29).

Independence in instrumental activities of daily living indicates that an older adult has not reached the frailty state. Another factor to consider is collateral circulation integrity (30). While studies suggest that collateral robustness declines with age, variability exists, and some older adult patients maintain robust collateral networks that may support favorable outcomes post-EVT. Advanced imaging modalities such as CT perfusion and collateral scoring could further refine patient selection by identifying older individuals who are more likely to benefit from intervention (31).

Our study identified higher baseline NIHSS and the absence of intravenous thrombolysis as the most influential predictors of poor post-EVT outcomes. This finding is consistent with prior literature demonstrating that initial stroke severity exerts a profound impact on EVT outcomes. Higher NIHSS scores are indicative of larger infarcts, increased neurological deficits, and lower likelihood of rapid functional recovery, particularly in older patients with reduced neuroplasticity. Prior studies, such as those by Inoue et al. (9) and Elsaid et al. (32) have confirmed that high NIHSS scores at presentation are independent predictors of poor EVT outcomes, particularly in patients aged ≥90 years (9, 32).

Our findings also emphasize the importance of intravenous thrombolysis as an adjunctive therapy in eligible patients. Recent trials, including the SWIFT-DIRECT and SKIP studies, suggest that bridging therapy with intravenous thrombolysis may enhance recanalization rates and functional recovery in select patient populations (33, 34). The results from these studies support the need for further investigation into optimizing combined treatment strategies in older patients undergoing EVT. In the foreseeable future, emerging thrombectomy technologies and refined procedural strategies are likely to further reshape the landscape of EVT. In particular, challenging cases of AIS due to distal vessel occlusions—such as M4 segment occlusions—may become more effectively manageable with advanced microcatheter-based aspiration techniques (35). In our cohort, a combined TAS + SR approach was employed in most patients. Notably, a suction-first (thromboaspiration-first) strategy appeared to be as safe in octogenarians and nonagenarians as in septuagenarians, without evidence of excess procedural complications. However, definitive conclusions regarding comparative safety and efficacy across advanced age groups require validation in adequately powered prospective randomized trials.

A major strength of this study is our focus on an older adult cohort, stratified into septuagenarians and octo/nonagenarians, which provides a more nuanced understanding of EVT outcomes in patients aged ≥70 years. BLR was employed to enhance the stability of effect estimates in a probabilistic framework, enabling more robust identification of key predictors associated with favorable functional outcomes. Additionally, the study reflects real-world EVT practice outside of randomized controlled trial settings, enhancing its generalizability. However, some limitations of our study should be acknowledged. First, this was a single-center study with a small sample size, which may limit the external validity. Second, the retrospective nature of the study introduces potential selection bias, as patients deemed too frail or with extensive infarcts may not have undergone EVT. The importance of age stratification in EVT research was also emphasized by Enriquez et al. (6), who found that functional outcomes declined by 8% per additional year of age in an older adult cohort of patients with EVT. Future studies incorporating baseline frailty assessments and quality-of-life measures could provide a more comprehensive evaluation of EVT candidacy in older adults.

## Conclusion

Our findings reaffirm that age alone should not be used as a strict exclusion criterion for EVT in older patients with AIS. Despite the intrinsic mortality risk associated with increasing age, our BLR analysis demonstrated that stroke severity and absence of intravenous thrombolysis—not age—were the primary determinants of poor outcomes. Careful patient selection based on functional status, collateral integrity, and individualized risk assessment remains crucial to optimize EVT success in older adult populations.

## Data Availability

The raw data supporting the conclusions of this article will be made available by the authors, without undue reservation.
