# Supplementary material for: Age demonstrates limited predictive utility for functional outcomes after thrombectomy in patients aged ≥70 years with acute ischemic stroke: a single-center cohort study
Source: Front Neurol. 2026 Apr 2;17:1772084. doi: 10.3389/fneur.2026.1772084 (PMC13082929; doi:10.3389/fneur.2026.1772084)
Supplement: Supplementary file 1 [file Supplementary_File_1.docx]

**Supplementary materials**

**Supplementary Table S1**. Proportion of older adults aged ≥70 years in the real-world registry databases

| **Registry^Ref^** | **Country** | **Study Period** | **Older Adult Proportion** |
| --- | --- | --- | --- |
| The Quality Improvement and Clinical Research (QuiCR) Registry^Zerna 2020^ | Alberta, Canada | 2015/4/1 to 2018/3/31 | **50%** aged **≥70**  **25%** aged **≥80** (approximation) |
| Multicenter Randomized Clinical Trial of Endovascular Treatment for Acute Ischemic Stroke in the Netherlands (MR CLEAN) Registry^Groot 2020^ | Netherlands | 2014 to 2016 | **25%** aged **≥80** |
| Get with the Guidelines-Stroke (GWTG-Stroke) program^Adcock 2022^ | American Heart Association/American Stroke Association, USA | 2012/4/1 to 2019/6/30 | **39.43%** aged ≥80; median [IQR] = 85 [82–89] |
| The Oslo Acute Reperfusion Stroke Study^Enriquez 2024^ | Norway | 2017/1/1 to 2022/7/31 | **21.2%** aged ≥80 |
| The Endovascular Treatment With versus Without Tirofiban for Stroke Patients with LVO (RESCUE BT) trial and the Direct Endovascular Thrombectomy versus Combined intravenous thrombolysis and Endovascular Thrombectomy for Patients With Acute Large Vessel Occlusion in the Anterior Circulation (DEVT) trial^Zhai 2023^ | China | 2018/5/1 to 2021/10/31 | **13.03%** aged ≥80 |
| The Tokyo/Tama-REgistry of Acute endovascular Thrombectomy (TREAT) Study^Inoue 2022^ | Tokyo, Japan |  | **8.16%** aged **≥90** |
| The Heidelberg Recanalization Registry (HeiReKa) database^Ippen 2024^ | Germany | 1999 to 2021 | **6.10%** aged **≥90** |

LVO: large-vessel occlusion. Numbers in bold are presented solely for emphasis.

**Supplementary Table S2**. Demographic and clinical characteristics stratified by three age groups in older patients undergoing endovascular thrombectomy

| **Variable** | **70–79 Age Group (n = 44)** | | **80–89 Age Group (n = 40)** | | **≥ 90 Age Group (n = 10)** | | ***p***-**value** |
| --- | --- | --- | --- | --- | --- | --- | --- |
| **Sex** |  | |  | |  | | 0.3756 |
| Male (%) | 22 (50%) | | 14 (35%) | | 4 (40%) | |  |
| Female (%) | 22 (50%) | | 26 (65%) | | 6 (60%) | |  |
| **NIHSS** |  | |  | |  | |  |
| Median (IQR) | 19 (9.75–22) | | 21.5 (17–24) | | 19 (15.5–22) | | 0.1104 |
| **DTP (min)** |  | |  | |  | |  |
| Median (IQR) | 128 (57–165.25) | | 127.5 (83–167.5) | | 121.5 (100–152.5) | | 0.9930 |
| **PTR (min)** |  | |  | |  | |  |
| Median (IQR) | 30.5 (25–39) | | 33.5 (24.5–48.25) | | 30.5 (26.5–47.25) | | 0.5374 |
| **ASPECTS** |  | |  | |  | |  |
| Median (IQR) | 8 | (7–9) | 7 | (5–9) | 6.5 | (5–8) | **0.0321** |
| **CTP infarct core (mL)** |  |  |  |  |  |  |  |
| Median (IQR) | 4 | (0–26) | 20.5 | (3–54.25) | 9.5 | (1.5–47.5) | 0.1258 |
| Not obtainable | 6 | 13.6% | 4 | 10.0% | 0 | 0.0% |  |
| **Occlusion site** |  |  |  |  |  |  | 0.6477 |
| A2 | 1 | 2.3% | 0 | 0% | 0 | 0% |  |
| ACA | 1 | 2.3% | 1 | 2.5% | 0 | 0% |  |
| ICA | 10 | 22.7% | 15 | 37.5% | 2 | 20.0% |  |
| M1 | 18 | 40.9% | 14 | 35.0% | 3 | 30.0% |  |
| M2 | 11 | 25.0% | 6 | 15.0% | 3 | 30.0% |  |
| Tandem | 3 | 6.8% | 4 | 10.0% | 2 | 20.0% |  |
| **Post-procedure TICI** |  |  |  |  |  |  | 0.7433 |
| 0 | 1 | 2.3% | 0 | 0% | 0 | 0% |  |
| 1 | 1 | 2.3% | 1 | 2.5% | 1 | 10.0% |  |
| 2a | 1 | 2.3% | 0 | 0% | 0 | 0% |  |
| 2b | 15 | 34.1% | 15 | 37.5% | 5 | 50.0% |  |
| 2c | 8 | 18.2% | 3 | 7.5% | 0 | 0% |  |
| 3 | 18 | 40.9% | 21 | 52.5% | 4 | 40.0% |  |
| **Hypertension** |  |  |  |  |  |  | 0.8070 |
| Y | 35 | 79.5% | 31 | 77.5% | 7 | 70.0% |  |
| N | 9 | 20.5% | 9 | 22.5% | 3 | 30.0% |  |
| **Type 2 diabetes** |  |  |  |  |  |  | 0.6096 |
| Y | 24 | 54.5% | 23 | 57.5% | 4 | 40.0% |  |
| N | 20 | 45.5% | 17 | 42.5% | 6 | 60.0% |  |
| **Hyperlipidemia** |  |  |  |  |  |  | 0.2222 |
| Y | 17 | 38.6% | 23 | 57.5% | 5 | 50.0% |  |
| N | 27 | 61.4% | 17 | 42.5% | 5 | 50.0% |  |
| **Atrial fibrillation** |  |  |  |  |  |  | 0.2621 |
| Y | 28 | 63.6% | 28 | 70.0% | 9 | 90.0% |  |
| N | 16 | 36.4% | 12 | 30.0% | 1 | 10.0% |  |
| **Coronary artery disease** |  |  |  |  |  |  | **0.0419** |
| Y | 16 | 36.4% | 17 | 42.5% | 8 | 80.0% |  |
| N | 28 | 63.6% | 23 | 57.5% | 2 | 20.0% |  |
| **Peripheral arterial obstructive disease** |  |  |  |  |  |  | 0.7445 |
| Y | 5 | 11.4% | 6 | 15.0% | 2 | 20.0% |  |
| N | 39 | 88.6% | 34 | 85.0% | 8 | 80.0% |  |
| **Cigarette smoking** |  |  |  |  |  |  | 0.2135 |
| Y | 9 | 20.5% | 3 | 7.5% | 1 | 10.0% |  |
| N | 35 | 79.5% | 37 | 92.5% | 9 | 90.0% |  |
| **Symptomatic ICH** |  |  |  |  |  |  | 0.9494 |
| Y | 10 | 22.7% | 8 | 20.0% | 2 | 20.0% |  |
| N | 34 | 77.3% | 32 | 80.0% | 8 | 80.0% |  |
| **mRS at 3 mo. post-EVT** |  |  |  |  |  |  | 0.0885 |
| 0 | 1 | 2.3% | 1 | 2.5% | 1 | 10.0% |  |
| 1 | 4 | 9.1% | 2 | 5.0% | 0 | 0.0% |  |
| 2 | 7 | 15.9% | 0 | 0.0% | 0 | 0.0% |  |
| 3 | 7 | 15.9% | 8 | 20.0% | 1 | 10.0% |  |
| 4 | 11 | 25.0% | 11 | 27.5% | 1 | 10.0% |  |
| 5 | 7 | 15.9% | 5 | 12.5% | 1 | 10.0% |  |
| 6 | 7 | 15.9% | 13 | 32.5% | 6 | 60.0% |  |
| **mRS at 3 months post-EVT, grouped** |  |  |  |  |  |  | **0.0124** |
| 0–2 (Good) | 12 | 27.3% | 3 | 7.5% | 1 | 10.0% |  |
| 3–5 | 25 | 56.8% | 24 | 60.0% | 3 | 30.0% |  |
| 6 (Death) | 7 | 15.9% | 13 | 32.5% | 6 | 60.0% |  |
| **mRS at 3 months post-EVT** |  |  |  |  |  |  | 0.1942 |
| 0–3 | 19 | 43.2% | 11 | 27.5% | 2 | 20.0% |  |
| 4–6 | 25 | 56.8% | 29 | 72.5% | 8 | 80.0% |  |
| **mRS at 3 months post-EVT** |  |  |  |  |  |  | **0.0453** |
| 0–2 (Good) | 12 | 27.3% | 3 | 7.5% | 1 | 10.0% |  |
| 3–6 | 32 | 72.7% | 37 | 92.5% | 9 | 90.0% |  |
| **Intravenous thrombolytic therapy** |  |  |  |  |  |  | 0.3351 |
| Y | 13 | 29.5% | 8 | 20.0% | 1 | 10.0% |  |
| N | 31 | 70.5% | 32 | 80.0% | 9 | 90.0% |  |
| **Family support** |  |  |  |  |  |  | 0.1498 |
| Y | 36 | 81.8% | 37 | 92.5% | 10 | 100.0% |  |
| N | 8 | 18.2% | 3 | 7.5% | 0 | 0.0% |  |
| **Fazekas grading of leukoaraiosis** |  |  |  |  |  |  | 0.0972 |
| 0 | 5 | 11.4% | 0 | 0.0% | 0 | 0.0% |  |
| 1 | 16 | 36.4% | 15 | 37.5% | 3 | 30.0% |  |
| 2 | 16 | 36.4% | 13 | 32.5% | 2 | 20.0% |  |
| 3 | 7 | 15.9% | 12 | 30.0% | 5 | 50% |  |

Bold typeface *p*-values in bold indicate statistical significance.

EVT: endovascular thrombectomy; ICH: intracerebral hemorrhage; PAOD: peripheral arterial occlusive disease; TICI: Thrombolysis in Cerebral Infarction grading system; CTP: CT perfusion; ASPECTS: Alberta Stroke Program Early CT Score; ICA: internal carotid artery; ACA: anterior cerebral artery; mRS: modified Rankin Scale; NIHSS: National Institutes of Health Stroke Scale. DTP: door-to-puncture time; PTR: Puncture-to-Reperfusion time

**Supplementary Table S3.** Sensitivity analysis accounted for the ischemic core in the Bayesian logistic regression in predicting good functional outcome in terms of mRS (0─2) at 3 months after endovascular thrombectomy.

| **Predictor** | **Odds ratio** | **Std. dev.** | **95% CrI** | **Median** | **MCSE** |
| --- | --- | --- | --- | --- | --- |
| Age | 0.96* | 0.04 | 0.88–1.04 | 0.96 | 0.01 |
| Intravenous thrombolysis | 9.01 | 8.15 | 1.54–30.74 | 6.56 | 0.39 |
| NIHSS | 0.91 | 0.05 | 0.82–0.996 | 0.91 | 0.003 |
| ASPECTS | 1.32* | 0.30 | 0.89–2.02 | 1.28 | 0.03 |
| CTP Infarct core | 0.97* | 0.02 | 0.93–1.005 | 0.97 | 0.001 |

*Because the 95% credible interval (CrI) includes 1, the posterior distribution indicates non-negligible uncertainty regarding the effect.

Odds ratio: Posterior mean odds ratio; ASPECTS: Alberta Stroke Program Early CT Score; CrI: credible interval; MCSE: Monte Carlo Standard Error; mRS: modified Rankin Scale; NIHSS: National Institutes of Health Stroke Scale; OR: odds ratio; Std. dev.: posterior standard deviation of the estimated parameter.

It is noteworthy that, prior to performing the Bayesian logistic regression, ten missing values in the variable computed tomography perfusion infarct core were imputed using K-nearest neighbors imputation with K = 5.

**Supplementary Table S4.** Posterior Probability of Effect Derived From Bayesian Logistic Regression In Predicting Good Functional Outcome (mRS 0–2) at 3 Months Post‑EVT.

| **Predictor** | **Adjusted Odds Ratio (aOR)** | **95% Credible Interval** | **Posterior SD** | **Posterior Probability of Effect** | **Interpretation** |
| --- | --- | --- | --- | --- | --- |
| **NIHSS** | 0.90 | 0.81–0.98 | 0.04 | 0.996 (β < 0) | Strong evidence of decreased odds |
| **ASPECTS** | 1.44 * | 0.97–2.08 | 0.30 | 0.887 (β > 0) | Moderate–strong evidence of benefit |
| **Age** | 0.95 * | 0.86–1.05 | 0.05 | 0.846 (β < 0) | Moderate evidence of decreased odds |
| **Intravenous Thrombolysis** | 6.59 | 1.16–23.09 | 5.62 | 0.631 (β > 0) | Weak–moderate evidence of benefit |

**Because the 95% credible interval (CrI) includes 1, the posterior distribution indicates non-negligible uncertainty regarding the effect.

Note: Posterior probability of effect represents the Bayesian probability that the parameter is in the stated direction (e.g., 𝑃 (𝛽 < 0) for OR < 1). In Bayesian terms, probabilities ≥0.95 typically indicate strong evidence.
